# Use of Cracker Residue in the Diet of Dairy Heifers: Impacts on Animal Health, Ruminal Fatty Acids Profile, Digestibility, Weight Gain, and Economic Viability

**DOI:** 10.3390/ani14091325

**Published:** 2024-04-29

**Authors:** Maksuel Gatto de Vitt, Aline Luiza do Nascimento, Andrei Lucas Rebelatto Brunetto, Arthur Mocelin Piaia, Charles Marcon Giocomelli, Ana Carolina Xavier, Roger Wagner, Camila Soares Martins, Gilberto Vilmar Kozloski, Aleksandro Schafer Da Silva

**Affiliations:** 1 Graduate Program in Animal Science, Universidade do Estado de Santa Catarina (UDESC), Chapecó 89815-000, SC, Brazil; maksuel.gdv99@edu.udesc.br (M.G.d.V.); aline.nascimento@edu.udesc.br (A.L.d.N.); andrei.brunetto@edu.udesc.br (A.L.R.B.); charles.giacomelli0864@edu.udesc.br (C.M.G.); 2 Department of Animal Science, Universidade do Estado de Santa Catarina (UDESC), Chapecó 89815-000, SC, Brazil; 09176809951@edu.udesc.br; 3 Graduate Program in Food Science, Federal University of Santa Maria (UFSM), Santa Maria 97105-900, RS, Brazil; anacarolinahadlich5@gmail.com (A.C.X.); rogerwag@gmail.com (R.W.); 4 Department in Animal Science, Federal University of Santa Maria (UFSM), Santa Maria 97105-900, RS, Brazil; camila.martins@acad.ufsm.br (C.S.M.); gilberto.kozloski@ufsm.br (G.V.K.)

**Keywords:** dairy cattle, nutrition, by-product

## Abstract

**Simple Summary:**

Cracker residue is a co-product of the food industry which has already been used in the diet of non-ruminants, but with little research for ruminants. Experiment I: Cracker residue can be used in feeding heifers, replacing 40% of the corn feed on an isometric scale (kg for kg). Experiment II: With the cracker residue it is possible to replace 100% of the corn feed, formulating a concentrate with the same energy and protein values. In both experiments, we highlighted discrete changes in the profile of voltaic fatty acids in the rumen, as well as those related to metabolism, which is directly related to the different composition of the ingredients used in the diet. Furthermore, the substitution of cracker residue did not negatively affect health and weight gain, and reduced the cost of the concentrate, consequently reducing the cost of production of these animals.

**Abstract:**

This study determined whether the isomeric or isoenergetic/isoproteic substitution of corn in the diet of Jersey heifers in the rearing phase with cracker residue would impair growth and health, as well as reducing production costs. Fourteen Jersey females in the growth phase were used, separated into two treatments with seven animals in each lot in collective pens. The experiment used 7-month-old animals (169.8 ± 2.89 kg) and lasted for four months. In Experiment I, the animals were divided into two groups: treatment, with the partial replacement of 40% corn with cracker residue, and control, in which the animals consumed the same diet with 100% corn (isometric diet kg for kg). In Experiment II, the animals with a body weight of 200.2 ± 3.85 kg were divided into two groups: Treatment, replacing 100% of the corn with cracker residue, and control, in which the animals consumed an isoprotein and isoenergetic diet but with 100% of the corn in the formulation. The diet consisted of concentrate, Tifton 85 hay, and corn silage, supplied twice a day individually, with animals contained in their feeders by kennels. There was water ad libitum in the bay. Biweekly weighing and monthly blood analysis were performed, totaling four collections per part for hematologic evaluation, carbohydrate, lipid, and protein metabolism variables. At the end of each experiment, ruminal fluid was collected to measure the volatile fatty acid profile, and feces were collected to determine the apparent digestibility coefficient (ADC). Experiments I and II showed no effect of treatment on body weight, weight gain, average daily weight gain, feed intake, and feed efficiency. There was no effect of treatment on leukocyte, erythrocyte, lymphocyte, neutrophil, monocyte, and eosinophil counts, hematocrit, and hemoglobin concentration (*p* > 0.05). Experiment I showed a difference between groups for the variables albumin, globulin, total proteins, cholesterol, glucose, and urea, which did not happen in Experiment II. In both experiments, a higher ADC of nutrients was found in the treatment group which had cracker residue (*p* > 0.05). The concentration of volatile fatty acids in Experiment I was higher in the control group, unlike in Experiment II, where the highest concentration was in the treatment group (*p* > 0.05). Because experiment I had an isometric substitution, the diets had different bromatological composition, which is the probable cause of the difference between groups; this did not happen in experiment II, in which the diets consumed by the animals was isoproteic and isoenergetic. Based on these data we conclude that the substitution of cracker residue in an isomeric or isoenergetic/isoproteic form does not negatively affect weight gain and animal health, as well as reduces the cost of the concentrate, consequently reducing the cost of production of these animals.

## 1. Introduction

The production of heifers is of extreme importance in dairy production. Cows with problems that affect production must be discarded to obtain a genetic improvement of the herd; this occurs in young animals, which have better productive activity [1]. According to Santos [2], the ideal culling rate is 20 to 30% of the lactating herd per year; therefore, many heifers are needed for a replacement rate above 25%. To obtain the desired indices, dairy heifers must be provided with quality feed, as their development is negatively affected by foods of low nutritional value due to the reduction in consumption and the nutritional value itself [3]. In the growth phase, it is possible to obtain significant daily gains, depending on balanced nutrition, health status, and the phase of the heifer’s life, as it is essential to develop a functioning immune system to have greater performance [4]. Furthermore, a better well-being and health, nutritional, and reproductive management results in a more productive adult animal [5].

After weaning, the rearing phase requires substantial attention. In a system in which dairy heifers are raised in confinement, feeding involves corn silage and quality hay; in milk production lines, concentrate during the breeding phase is recommended [6]. When demand is high and inventory is low of corn, the price rises [7]. In southern states in the US, severe droughts affected corn productivity, and in the Midwest, the delay in the soybean harvest due to climatic factors delayed planting, which, combined with high demand from the livestock sector, caused an increase in the price of the grain [8]. For these reasons, the use of by-products in dairy cattle is an economically viable alternative to conventional feeds, especially for heifers [9].

Brazil is among the leading producers of crackers worldwide. According to Abimapi [10], in 2020, the country produced 1250 tons of crackers/biscuits. Garcia et al. [11] found that for every 70 tons of crackers produced, approximately five tons were lost during manufacturing. For a long time, this residue was discarded, but it then started to be used in animal feed thanks to research advances. According to Arosemena et al. [12], cracker residue is an energetic food due to the large amount of soluble carbohydrates. However, due to the low number of published works on the actual nutritional value for animals, its use is limited [13] or uncontrolled without technical guidance. Adams [14] found that a maximum of 10% of the dry matter of the total diet can be used, or 20% of the dry matter of the concentrate of the residue for lactating cows; however, at more significant levels, it can affect the fat content in the milk. This by-product could be used in more significant amounts for heifers and dry cows; however, there are few studies to establish the quantities to be used precisely.

The chemical composition of cracker residue is available in the Brazilian table for pigs and poultry [15], which allows the formulation for these species. However, for ruminants, this information is not available in a database of formulations for cattle and small ruminants. In Brazil, according to Rostagno et al. [15], cracker residue has 92.5% dry matter (DM) and has a chemical composition of 8.69% crude protein (CP), 46.5% starch, 4.36% neutral detergent fiber (NDF), 1.61% acid detergent fiber (ADF), 91.2% organic matter (OM), and 1.31% mineral matter, as well as 4341 kcal/kg of crude energy. Processing in the production of crackers, produced mainly from wheat flour, could increase the digestibility of this feed when compared to ground corn; in addition, it has a chemical composition closer to that of corn, which is the main ingredient in concentrates for animals. Our hypothesis was that cracker residue would indeed be an alternative feed to replace corn in ruminant feed, but only in a diet formulated to be isoenergetic and isoproteic would it not negatively affect weight gain and animal health (unlike the isomeric diet, which is hypothesized to harm the growth of calves as it is not a balanced diet). Therefore, the present study determined whether the isomeric or isoenergetic/isoproteic substitution of corn in the diet of Jersey heifers in the rearing phase with cracker residue would not impair growth and health, as well as reducing production costs.

## 2. Materials and Methods

### 2.1. Cracker Residue and Other Concentrate Ingredients

Cracker residue is a by-product of the food industry and was provided by Casaredo^®^ (São Lourenço do Oeste, SC, Brazil). The crackers were made with cornstarch without animal products. For the concentrate formulation, a prior bromatological analysis of the residue was performed, and that of the other ingredients used for the formulation (Table 1).

In Experiment I, cracker residue replaced 40% of the corn. In Experiment II, the cracker residue replaced 100% of the corn. In addition to the inclusion ratios, Experiments I and II differed in the form of substitution: an isometric (kg/kg ratio) formulation in experiment I and isoproteic and isoenergetic formulations (same protein and energy value) in experiment II.

### 2.2. Animals and Installations

The study was performed at the UDESC Experimental Farm (FECEO) in Guatambu/SC, in the ruminant sector, for four months. In the two experimental periods the females were housed in adequate facilities in a collective pen of 250 m^2^ (10 × 25 m) for 14 animals, which had individual feeders controlled by kennels and a drinker with a float to control the water flow.

The study involved Jersey heifers in the growth phase, with an average age of seven months and an average initial weight of 169.8 ± 2.89 kg. The animals in this research were purchased from rural producers in the region, raised in the experimental station since they were 30 days old, and have already been used in other research at the experimental farm of Universidade do Estado de Santa Catarina (UDESC), approved by the ethics committee on the use of animals of the university (protocol number 4985190421).

### 2.3. Experiment I: Experimental Design and Diets

Experiment I lasted 60 days (15 days of adaptation + 45 days of data collection), in which the animals were randomly divided into two groups of seven heifers: Treatment, in which the concentrate had a replacement of 40% of the corn by cracker residue (alternative feed), and Control, in which the concentrate had corn, the traditional feed.

The diets were formulated individually for each animal according to the nutritional requirements of the animals [16], considering the following feeds: concentrate, Tifton 85 hay, and corn silage mixed and supplied in individual feeders, divided into two daily feeds. It is essential to mention that in Experiment I, the replacement was isometric: For every 1 kg of ground corn removed from the formulation, 1 kg of crushed cracker residue was added. The supply of water was ad libitum. The composition of the concentrate and the feeds used in the diet are shown in Table 1.

### 2.4. Experiment II: Experimental Design and Diets

After completing Experiment I, there were 15 days when all animals consumed the same diet based on corn silage, hay, and control concentrate. At the end of this period, the animals were again randomly distributed into two groups, when they were 9 months old and had a BW of 200.2 ± 3.85 kg. Experiment II lasted 60 days (15 days of adaptation + 45 days of data collection), in which the animals were randomly divided into two groups of seven heifers: Treatment, in which the concentrate used cracker residue in a proportion of 100% replacing ground corn, and control, in which the concentrate used 100% corn (traditional feed). The concentrates were formulated to be isoproteic and isoenergetic, with the same values for protein and energy. Because the residue has higher levels of ether extract, it was necessary to use soybean oil in the control concentrate to balance the energy values.

The diets were formulated individually for each animal group according to the nutritional requirements of the animals [16]. The feeds used were concentrate, Tifton 85 hay, and corn silage, mixed and supplied in individual feeders, divided into two daily feeds. The water supply was ad libitum. The composition of the concentrate and the feeds used in the diet are shown in Table 1.

### 2.5. Experiments I and II: Feed Management

The Jersey females received twice a day (08:00 h and 16:00 h) a total mixed ration (TMR) based on corn silage, hay, and concentrate in individual feeders. The animals were trapped for 90 min in the morning and 90 min in the afternoon, totaling 3 h per day for feeding. After this period, the animals are kept in a collective pen with free access to water. If there was food left over, it was weighed and recorded.

### 2.6. Experiments I and II: Growth Performance

The heifers were individually weighed five times (days 1, 15, 30, 45, and 60 of the experiment). All weighings were performed in the morning, with the heifers fasting for 12 h, with a digital electronic bar scale (DIGITRON^®^, ULB-300-90CM, Patos de Minas, MG, Brazil). Feed consumption was calculated based on the quantity supplied, subtracting the quantity left over. With the weight data of each collection, it was possible to calculate the weight gain (WG) (WG = current collection weight − previous collection weight) and average daily gain (ADG = (day 15 weight − day 60 weight)/number of days)). From the results of WG and daily feed consumption, it was possible to calculate feed efficiency.

### 2.7. Experiments I and II: Sample Collection

Blood collections were performed on days 1, 15, 30, and 60, totaling four collections in each experiment. Blood collection was carried out in the morning (06:00 h), with animals that had been consuming feed for approximately 12 h. Samples were collected in vacuolated tubes. Tubes with anticoagulants were used for complete blood count, and for serum, tubes without anticoagulants were used. After collection, these samples were sent to the laboratory and centrifuged at 2800× *g* for 10 min to separate serum and plasma. Then, fractions of serum and plasma were stored in 1.8 mL Eppendorf microtubes at −15 °C.

At the end of each experiment (day 60), ruminal fluid collection was standardized precisely four hours after the morning feeding [17]. Ruminal fluid was collected with an esophageal probe coupled to a suction motor. The pH of the rumen fluid was then measured using a portable digital pH meter (Testo 205). Part of the remaining rumen fluid was filtered through three gauzes and stored in 3 mL microtubes (Eppendorf^®,^ São Paulo, Brazil) and frozen at −20 °C for subsequent analysis of volatile fatty acids (VFA).

### 2.8. Laboratory Analysis

#### 2.8.1. Analysis of the Conventional Chemical Composition of Feed and Feces

In the laboratory, the samples were pre-dried in a forced ventilation oven at 55 °C for 72 h, then removed from the oven and weighed again to determine the partial dry matter content, followed by grinding in a Wiley-type mill (model: MA340, Marconi, Brazil), using a 1 mm mesh sieve. The pre-dried and ground samples were heated at 105 °C to obtain the dry matter and the mineral material in a muffle at 600 °C [18]. The micro-Kjeldahl method determined the nitrogen content (Method 984.13) [19], which allowed for predicting the crude protein content through mathematical calculation (TP in g/100 g = (Va − Vb) × fa × F × 0.14)/P). To determine the neutral detergent fiber (NDF) content, the samples were placed in polyester bags [20] and treated with a neutral detergent solution in an autoclave at 110 °C for 40 min [21]; for concentrate samples, we included α-amylase. Acid detergent fiber (ADF) concentrations were determined according to AOAC [19] (method 973.18). Values were obtained using a Near Infrared Reflectance Spectrometer (NIRS), model Spectra Star 2600 XT series of Near Infrared Analyzers (Unity Scientific^®^, New York, NY, Unites States) for starch, carbohydrates, and ether extract. It was not possible to perform NDF and FDA analysis of the concentrate ingredients individually, and starch and carbohydrate analysis was not possible via NIRS on silage, hay, and TMR. The results are shown in Supplementary Materials Table S1 and Table 1.

#### 2.8.2. Apparent Digestibility

In the final three days of the study [22,23], fecal samples (*n* = 7 per group) were collected directly from the rectal ampulla at days 58, 59, and 60 of Experiments I and II (8:00 h, 12:00 h, and 16:00 h). After collection, they were dried in a forced ventilation oven (55 °C by 72 h). The samples were processed in a specific mill (Wiley^®^ Mill, Curitiba, Brazil) with a 1 mm sieve and stored for analysis.

Indigestible neutral detergent fiber (iNDF) was used to determine apparent digestibility, as described by researchers [24]. The feed and feces samples were incubated in bovine rumen for 288 h, washed, and dried in a forced ventilation oven (55 °C by 72 h). NDF and ADF concentrations were determined to calculate digestibility [25].

#### 2.8.3. Hematologic Analysis

The blood count was performed within a maximum of 2 h after collection. Blood samples with anticoagulant and a semi-automatic blood cell contactor (CELM CC530, Barueri/SP, Brazil) were used. Subsequently, a blood smear was used to perform the differential leukocyte count using a light microscope with a magnification of ×1000. Hematocrit was determined using microcapillaries and centrifugation (10,000 rpm for 5 min), with subsequent reading using a standard centrifuge card.

#### 2.8.4. Serum Biochemistry

Serum levels of total protein, albumin, cholesterol, triglycerides, glucose, and urea were evaluated, and the activity of the enzymes aspartate aminotransferase (AST) and gamma-glutamyl transpeptidase (GGT) in serum was determined using commercial kits (Analisa, Belo Horizonte/MG, Brazil) and semi-automatic equipment (BIO PLUS 2000^®^, Barueri/SP, Brazil), according to the manufacturers’ recommendations. Globulin levels were obtained using globulin = total protein − albumin.

#### 2.8.5. Profile of Fatty Acids in Ruminal Fluid: Experiments I and II

Ruminal fluid samples were thawed until they reached a temperature of 5 °C and then manually homogenized. After these processes, 1 mL aliquots of the supernatant of ruminal fluid samples were transferred to polypropylene microtubes (2 mL), which were subsequently centrifuged for 5 min (12,300× *g*). Then, 100 μL of the supernatant was removed and transferred to a new microtube containing 100 μL of formic acid. The mixture was vortexed for 30 s and centrifuged again for 3 min. After centrifugation, 50 μL of the supernatant of the mixture was transferred to 250 μL tubes, and 100 μL of the methanolic solution of the internal standard 3-octanol (665 μg mL^−1^) was added. Samples were injected into a gas chromatograph with a flame ionization detector (GC-FID; Varian Star 3400) and an autosampler (Varian 8100). One microliter of the extract was injected in a 1:10 split mode. The carrier gas used was hydrogen at a constant pressure of 20 psi. The analytes (acetic, propionic, butyric, valeric, and isovaleric acids) were separated on a CP WAX-52CB capillary column (60 m × 0.25 mm; 0.25 μm stationary phase thickness). The initial temperature of the column was 80 °C for 1 min and increased by 15 °C min^–1^, reaching 120 °C, then reaching 230 °C rising by 20 °C min^–1^, where it remained for 1 min. The injector and detector temperatures were set to 250 °C. Method validation comprised the following parameters: selectivity, linearity, linear range, repeatability, precision, limit of detection (LOD), and limit of quantification (LOQ) for acetic, propionic, butyric, valeric, and isovaleric acids. The analytical parameters are presented in Supplementary Materials Table S1. Linearity was assessed by calculating a regression equation using the least squares method. LOD and LOQ values were achieved by sequential dilutions up to signal-to-noise ratios of 3:1 and 6:1, respectively. Precision was assessed by analyzing the repeatability of six replicated samples. Accuracy was determined by recovering known amounts of standard substances added to the samples. The results were expressed in mmol L^−1^ of each VFA in ruminal fluid.

### 2.9. Economic Viability

To analyze the economic viability, the value of the concentrates was calculated according to the prices and quantity of the ingredients used: corn (0.30 $/kg), soybean bran (0.42 $/kg), soybean hulls (0.32 $/kg), wheat bran (0.32 $/kg), soybean oil (1.44 $/kg), mineral core (0.99 $/kg), and cracker residue (0.037 $/kg). The value of the cracker residue was determined by the company that sold it, while the other ingredients were purchased in local shops. The roughages were produced in the experimental farm, considering the cost of corn silage (0.092 $/kg) and Tifton 85 hay (0.185 $/kg). Values may vary according to each region. To determine economic viability, information on feed consumption during the trial period, total diet cost, and WG during the trial period was used to determine the production costs to achieve 1 kg of animal body weight.

### 2.10. Statistical Analyses

A completely randomized design experiment was used, with two groups and seven replications each. Data were tested for normality and homogeneity of variance using Shapiro–Wilk and Levene tests, respectively. All data were analyzed using the SAS “MIXED procedure” (SAS Inst. Inc., Cary, NC, USA; version 9.4), with Satterthwaite approximation to determine denominator degrees of freedom for the fixed effects test. Diet digestibility and growth performance data were tested for fixed-effect treatment using animal (treatment) as a random effect. Weight, blood count, serum biochemistry, and ruminal fatty acid data were analyzed as repeated measures and tested for treatment fixed effects and treatment x day using animal (treatment) random effects. All data obtained on day 1 for each variable were included as covariates in each respective analysis. According to the lowest Akaike information criterion, the first-order autoregressive covariance structure was selected first. Averages were separated using the PDIFF method (T-test), and all results were reported as LSMEANS followed by SEM (standard error mean). Significance was defined when *p* ≤ 0.05, and trend when *p* > 0.05 and ≤ 0.10.

## 3. Results

### 3.1. Performance

The results of growth performance are shown in Table 2. In Experiments I and II, no treatment effect was verified on body weight, average daily WG, feed intake, and feed efficiency (*p* > 0.05).

### 3.2. Apparent Digestibility

The chemical composition of feces differed between treatments (Table 3). The apparent digestibility of dry matter and crude protein was high in the treatments of experiment I (*p* > 0.05 and *p* < 0.10; Table 3), and NDF tended to be higher in the treatment group in Experiment I (*p* ≤ 0.05; Table 3). The apparent digestibility of dry matter, ash, and ether extract (EE) was major for heifers of treatment group in Experiment II (*p* ≤ 0.05; Table 3). ADF tended to be higher in the treatment group in Experiment II (*p* > 0.05 and *p* < 0.10; Table 3).

### 3.3. VFAs in Ruminal Fluid

Volatile fatty acid profile results are presented in Table 4. In Experiment I, the treatment effect was verified for acetic acid, propionic acid, butyric acid, and isovaleric acid (*p* ≤ 0.05). For these four VFAs, the lowest concentration in ruminal fluid was observed in treatment heifers compared to the control (*p* ≤ 0.05). In Experiment II, the treatment effect was verified for acetic acid, propionic acid, and butyric acid (*p* ≤ 0.05), and for these three VFAs, the highest concentration in the ruminal fluid was observed in the heifers of the treatment when compared to the control (*p* ≤ 0.05). Isovaleric acid was not affected by treatment in Experiment II (*p* > 0.05).

### 3.4. Biochemistry and Metabolism

The results of the serum biochemistry and metabolism of Experiment I are presented in Table 5. The treatment effect for albumin, globulin, total protein, urea, and glucose (*p* ≤ 0.05) was verified. Except for urea, which was lower in the serum of animals in the treatment group (*p* ≤ 0.05), the other variables mentioned above were higher in the treatment group than in the control group (*p* ≤ 0.05). The variables triglycerides, cholesterol, AST, and GGT had no treatment effect (*p* ≤ 0.05). Interaction between treatment vs. day was observed for albumin, globulin, glucose, and cholesterol, and for albumin, globulin, and cholesterol, the effect was observed on day 15 (the greatest in the treatment group) (*p* ≤ 0.05); for glucose, the interaction occurred on days 30 and 60, being more significant in the treated animals (*p* ≤ 0.05). The interaction trend for urea (days 30 and 60) was observed, being lower in the treatment group (*p* ≤ 0.05). There was no interaction between treatment vs. day (*p* ≤ 0.05) for the other variables (total protein, triglycerides, AST, and GGT).

The results of Experiment II for experimental biochemistry and metabolism are presented in Table 6. There was no treatment effect for any biochemical variable analyzed and no interaction between treatment and day (*p* > 0.05), except triglyceride levels, which were higher in the treatment group than the control on days 30 and 60 of Experiment II (*p* ≤ 0.05).

### 3.5. Hematology

The blood count results of Experiments I and II are presented in Supplementary Materials Tables S3 and S4. There was no treatment effect or interaction between treatment vs. day for white blood cell, erythrocyte, lymphocyte, neutrophil, monocyte, and eosinophil counts, and hematocrit and hemoglobin concentration (*p* > 0.05). In Experiment I (Supplementary Materials Table S3), the total white blood cell counts in the blood tended to be lower in the treatment compared to the control.

### 3.6. Economic Viability

In Experiment I, the control concentrate had a higher cost per kilogram (kg) than the treatment concentrate when replacing 40% with cracker residue, amounting to an increase of 15.1%. For each kg of live weight gained by the animal during the experiment, the control diet had a higher cost than the treatment diet, that is, it was 17.7% cheaper when using the cracker residue (Table 7). In Experiment II, the control concentrate cost 56% more than the treatment concentrate that contained cracker residue. For each kg of live weight that the animal gained in the experiment, the treatment diet cost was reduced by 28.2% compared to the diet with corn in the composition (Table 7).

## 4. Discussion

The 40% isometric and 100% isoproteic and isoenergetic substitutions did not affect growth performance. Passini et al. [26] substituted corn in a 30% isometric proportion for cracker residue in the diet of steers and found no loss in performance but reduced expenditure on feed. Oliveira et al. [27] substituted 20, 40, 60, and 80% of the corn by baking residue in diets of finishing sheep and found no adverse effect on growth. Adams [14] suggested that for heifers, this by-product in amounts greater than 10% of the dry matter of the total diet or 20% of the dry matter of the concentrate would not cause damage to animal growth. In both experiments, a higher ADC of nutrients was found in the treatment group, which had cracker residue. Our hypothesis is that the cracker residue is already a processed ingredient, with exposure to heat, a physical factor that tends to weaken nutrient binding and consequently favors absorption compared to ground corn, which is a hard feed.

Given the volatile fatty acids (VFAs) in Experiment I, the values indeed differed because there was no protein and energy balance in the diet, in which only 40% of one ingredient was replaced by another. In Experiment II, in which there was a diet with the same energy and protein values, there was no effect between treatments due to the consumption of cracker residue. Through the fermentation of carbohydrate and protein microorganisms, VFAs are produced in the rumen, which is the primary source of energy for ruminants [28]. The quantities and types of VFAs produced will depend on the type of feed, species of microorganisms present, and the ruminal environment in the fermentation process [29]. In our study, VFA levels differed between treatments, which seems related to the diets and the alternative ingredients tested here. The main VFAs produced in the rumen are propionic, acetic and butyric acids, which are sensitive biomarkers of nutrition being altered in our study. The changes were discrete, and probably have a direct relationship with the total mixed ration (TMR) and modulation of microorganisms in the ruminal environment.

The serum biochemistry and metabolism variables of Experiment I were higher in the treated group in some parameters compared to the control because the diets were not isoproteic or isoenergetic, thus causing a variation in the chemical composition of the diets. This result differs from Experiment II, in which the diets were equal in energy and protein content, with no relevant changes in the values found that remained within the reference values for the species [30], except for the levels of triglycerides that were higher than those found in the literature. The proportion of non-structural carbohydrates may interfere with the serum glucose level [31]. Diversified energy sources can increase the animal’s glucose availability [30]. In the industrialization of crackers, the ingredients undergo thermal treatment, compared to the extrusion process, making nutrients more available. This process can disrupt the matrix surrounding the starch granules, improving their digestibility [32]. Physical exercises or stress that the animals may be subjected to during the blood collection period can lead to variations in the serum glucose values [33]. However, we do not believe this is the reason because the alteration occurred only in Experiment I. GGT and AST activity did not change between groups, a positive result demonstrating that the use of cracker residue does not cause liver damage in animals. Alvarenga et al. [34] showed that AST activity oscillated between 73.05 and 102.41 U/L and GGT activity between 16.15 and 9.93 U/L for clinically healthy Jersey cows, similar to our work.

The blood counts in Experiments I and II showed no changes between treatments, demonstrating that the use of cracker residue in an isometric proportion of 40% and the use of 100% in an isoproteic and isoenergetic form concerning corn did not negatively affect the health of the animals, as it did not show low immunity, anemia, or malnutrition. The values were within the reference values for the Jersey breed [35,36]. The leukocyte count in Experiment I was high; according to Jain [37], leukocytosis may occur due to stressful management factors or high sanitary challenges. Both conditions contributed to this alteration in Experiment I as it took place during the Brazilian winter, and although the animals have a covered area for protection, a large part of the paddock is made of dirt, which, due to the frequent rains of this season, ends up generating clay.

During the work period, the cost of corn was $ 0.30/kg, and the residue was sold at $0.037/kg by the company, taking into account the oscillation and constant increase in the price of corn. In Experiment I, the value of the concentrated treatment was 15.1% lower than the control, yet for each kg of live weight gained, the diet that consisted of the partial replacement of 40% of corn cost 17.7% less than the diet with no replacement of corn. This value is equivalent to the study by Passini et al. [26], who, in the partial replacement of 30% of corn with cracker residue, showed a reduction of 56% in the diet. In Experiment II with total replacement of corn, the control concentrate had a cost 28.2% higher than the treated one; in addition, for each kg of weight gained in the experiment, the diet that had cracker residue had a cost reduction of 22.8% when compared to the control diet. In Gebert et al. [38], replacing cracker residue with corn in laying hens made it possible to reduce the cost of the diet by 22.3%, a value close to that found in our work.

## 5. Conclusions

Cracker residue can be used in feeding heifers, replacing 40% of the corn in an isometric way without negatively affecting the performance and health of the animals, despite statistical differences, as the variables remained within the reference limits. With the cracker residue, it is possible to replace 100% of the corn, formulating a concentrate with the same energy and protein values, having no effects on the growth and health of the heifers. The substitution of cracker residue reduces the cost of the concentrate, consequently reducing the cost of production of these animals.

## Figures and Tables

**Table 1:** Ingredients and chemical composition of feed (corn silage, hay, and concentrate) and total mixed ration (TMR).

Ingredients	g/kg					
Experiment I						
Corn silage	516.60					
Hay	115.00					
Concentrate	368.40					
Total diet	1000					
Chemical composition ^2^	Corn silage	Tifton hay	CONC-CON ^1^	CONC-TREAT ^2^	TMR-CON	TMR-TREAT
DM, %	36.76	81.26	87.68	88.11	41.20	42.70
Ash, %	3.21	4.66	5.54	5.40	5.40	5.17
CP, %	7.95	6.83	18.45	19.75	10.89	11.37
NDF, %	51.60	80.62	35.29	28.73	36.01	35.82
ADF, %	28.17	40.35	15.10	12.65	19.01	18.35
Starch	-	-	28.65	19.43	-	-
EE	-	-	2.66	4.39	2.87	3.21
SUGAR	-	-	10.26	17.10	-	-
Experiment II						
Corn silage	553.70					
Hay	84.70					
Concentrate	361.60					
Total diet	1000					
Chemical composition	Corn silage	Tifton hay	CONC-CON ^3^	CONC-TREAT ^4^	TMR-CON	TMR-TREAT
DM, %	36.76	81.26	85.63	89.68	48.60	51.25
Ash, %	3.21	4.66	5.27	6.08	6.28	5.91
CP, %	7.95	6.83	22.97	22.89	13.34	12.74
NDF, %	51.60	80.62	21.19	31.88	45.40	40.93
ADF, %	28.17	40.35	10.88	11.90	23.47	21.16
Starch, %	-	-	23.10	12.22	-	-
EE, %	-	-	4.74	6.09	3.30	3.70
SUGAR, %	-	-	12.46	23.62	-	-

^1^ Concentrate (CONC) composition: corn (400 g/kg), soybean bran (260 g/kg), soybean hulls (170 g/kg), wheat bran (110 g/kg) and 60 g/kg of mineral core. ^2^ Concentrate composition: corn (240 g/kg), cracker residue (160 g/kg), soybean bran (260 g/kg), soybean hulls (170 g/kg), wheat bran (110 g/kg) and 60 g/kg of mineral core. ^3^ Concentrate composition: corn (370 g/kg), soybean bran (290 g/kg), soybean hulls (170 g/kg), wheat bran (110 g/kg), soybean oil (30 g/kg) and 30 g/kg of mineral core. ^4^ Concentrate composition: cracker residue (405 g/kg), soybean bran (285 g/kg), soybean hulls (170 g/kg), wheat bran (110 g/kg), and 30 g/kg of mineral core. Note 1: Composition of mineral core: calcium min. 107 max. 132 g; phosphorus max. 88 g; sodium min. 126 g; sulfur min. 12 g; magnesium min. 9 g; cobalt min. 60 mg; iodine min. 75 mg; manganese min. 1300 mg; selenium min. 15 mg; zinc min. 3630 mg; fluorine max. 880 mg; iron min. 1800 mg; chromium min. 30 mg; vitamin A min. 20,000 IU; vitamin D3 min. 2500 IU; vitamin E min 350 IU. Note 2: DM (dry matter), MM (mineral matter), CP (crude protein), NDF (neutral detergent fiber), ADF (acid detergent fiber), and EE (ether extract).

**Table 2:** Growth performance of Jersey heifers fed (or not) with cracker residue.

Variables ^1^	Experiment I: Groups		SEM ^2^	*p*-Value
	Control	Treatment		
Initial weight *, kg	168.2	171.5	2.89	0.87
Final weight ^&^, kg	195.7	201.2	3.06	0.62
Average daily gain (ADG), kg/day	0.61	0.66	0.02	0.45
Dry matter intake (DMI), kg/day	3.21	3.26	0.18	0.95
Feed efficiency (ADG/DMI), kg/kg	0.19	0.20	0.01	0.61
**Variables ^1^**	**Experiment II: Groups**		**SEM ^2^**	***p*-Value**
	**Control**	**Treatment**		
Initial weight *, kg	203.5	197.2	3.85	0.79
Final weight ^&^, kg	233.3	228.1	3.14	0.76
Average daily gain (ADG), kg/day	0.63	0.64	0.03	0.87
Dry matter intake (DMI), kg/day	4.87	5.00	0.20	0.70
Feed efficiency (ADG/DMI), kg/kg	0.13	0.13	0.01	0.78

* corresponds to the end of the period of adaptation to the diet (day 15 of the experiment). ^&^ corresponds to the end of the experiment (day 60). ^1^ Treatments were: heifers that received a diet with cracker residue (treatment); animals that did not receive cracker residue in the diet (control). ^2^ SEM—standard error mean. Note: averages do not differ between groups (*p* ≤ 0.05) or tend to differ (*p* ≤ 0.10).

**Table 3:** Diet digestibility of Jersey heifers fed (or not) with cracker residue.

Variables ^1^	Experiment I		SEM ^2^	*p*-Value
	Control	Treatment		
DM	0.571	0.638	0.010	0.001
CP	0.472	0.556	0.009	0.001
NDF	0.480	0.534	0.010	0.054
ADF	0.513	0.520	0.011	0.896
EE	0.659	0.688	0.009	0.714
Ash	0.612	0.747	0.021	0.001
**Variables ^1^**	**Experiment II**		**SEM ^2^**	***p*-Value**
	**Control**	**Treatment**		
DM	0.494	0.549	0.009	0.050
CP	0.488	0.516	0.011	0.118
NDF	0.457	0.430	0.011	0.574
ADF	0.424	0.466	0.010	0.082
EE	0.561	0.628	0.008	0.002
Ash	0.519	0.752	0.023	0.001

^1^ Treatments were: heifers that received a diet with cracker residue (treatment); animals that did not receive cracker residue in the diet (control). ^2^ SEM—standard error mean. Note: averages do not differ between groups (*p* ≤ 0.05) or tend to differ (*p* ≤ 0.10).

**Table 4:** Profile of fatty acids in the ruminal fluid of Jersey heifers fed (or not) with cracker residue.

Variables ^1^	Experiment I		SEM ^2^	*p*-Value
	Control	Treatment		
pH	6.565	6.498	0.06	0.14
Total VFA, mmol L^−1^	65.89	50.31	3.02	0.01
Acetic acid, mmol L^−1^	48.2	37.6	2.74	0.01
Propionic acid, mmol L^−1^	8.61	6.24	0.81	0.05
Butyric acid, mmol L^−1^	8.21	5.87	0.92	0.02
Isovaleric acid, mmol L^−1^	0.87	0.60	0.05	0.01
**Variables ^1^**	**Experiment II**		**SEM ^2^**	***p*-Value**
	**Control**	**Treatment**		
pH	6.028	5.905	0.03	0.26
Total VFA, mmol L^−1^	40.79	58.10	3.52	0.01
Acetic acid, mmol L^−1^	30.6	42.9	3.21	0.01
Propionic acid, mmol L^−1^	5.10	7.15	0.28	0.01
Butyric acid, mmol L^−1^	4.43	7.31	0.42	0.01
Isovaleric acid, mmol L^−1^	0.66	0.74	0.07	0.39

Note: valeric acid was not detected. ^1^ Treatments were: heifers that received a diet with cracker residue (treatment); animals that did not receive cracker residue in the diet (control). ^2^ SEM—standard error mean. Note: averages do not differ between groups (*p* ≤ 0.05) or tend to differ (*p* ≤ 0.10).

**Table 5:** Experiment I: Serum biochemistry of Jersey heifers fed (or not) with cracker residue.

Variables ^1^	Treatments ^2^		SEM ^2^	*p*-Value	
	Control	Treatment		Treat	Treat × Day
Albumin (g/dL)				0.03	0.01
d 1	4.17	3.90	0.20		
d 15	4.53 ^b^	6.44 ^a^	0.19		
d 30	2.71	3.69	0.20		
d 60	3.37	4.17	0.20		
Globulin (g/dL)				0.01	0.01
d 1	6.50	6.02	0.23		
d 15	5.16 ^b^	9.06 ^a^	0.24		
d 30	4.47	4.89	0.23		
d 60	3.89	4.23	0.23		
Total protein (g/dL)				0.05	0.14
	8.04 ^b^	10.8 ^a^	0.29		
Urea (mg/dL)				0.05	0.07
d 1	18.2	18.1	0.10		
d 15	21.0	21.1	0.09		
d 30	27.5 ^a^	23.2 ^b^	0.11		
d 60	19.4 ^a^	16.2 ^b^	0.10		
Glucose (mg/dL)				0.05	0.01
d 1	122	116	11.74		
d 15	80.2	91.3	8.14		
d 30	83.1 ^b^	126 ^a^	8.17		
d 60	87.2 ^b^	109 ^a^	8.07		
Triglycerides (mg/dL)				0.59	0.44
	28.8	32.0	4.36		
Cholesterol (mg/dL)				0.11	0.01
d 1	151	138	6.74		
d 15	93.1 ^b^	131 ^a^	6.72		
d 30	68.2	74.3	6.59		
d 60	104	99.4	6.57		
AST (U/L)				0.82	0.79
	77.1	80.8	10.9		
GGT (U/L)				0.65	0.27
	12.8	16.9	4.00		

^1^ Treatments were: heifers that received a diet with cracker residue (treatment); animals that did not receive cracker residue in the diet (control). ^2^ SEM—standard error mean. Note: averages do not differ between groups (*p* ≤ 0.05) or tend to differ (*p* ≤ 0.10), identified by different letters (^a,b^) on the same line.

**Table 6:** Experiment II: Serum biochemistry of Jersey heifers fed (or not) with cracker residue.

Variables ^1^	Treatments ^2^		SEM ^2^	*p*-Value	
	Control	Treatment		Treat	Treat × Day
Albumin (g/dL)				0.96	0.92
	2.92	2.98	0.10		
Globulin (g/dL)				0.95	0.93
	3.94	3.80	0.10		
Total protein (g/dL)				0.91	0.82
	6.86	6.78	0.20		
Urea (mg/dL)				0.98	0.97
	21.0	21.9	0.12		
Glucose (mg/dL)				0.60	0.37
	69.5	75.0	5.89		
Triglycerides (mg/dL)				0.41	0.05
d 1	26.7	20.1	2.74		
d 15	24.0	22.2	2.79		
d 30	23.8 ^b^	30.0 ^a^	2.79		
d 60	22.5 ^b^	29.1 ^a^	2.75		
Cholesterol (mg/dL)				0.25	0.45
	105.7	96.5	4.51		
AST (U/L)				0.74	0.69
	62.8	58.7	6.40		
GGT (U/L)				0.80	0.15
	16.0	14.0	2.09		

^1^ Treatments were: heifers that received a diet with cracker residue (treatment); animals that did not receive cracker residue in the diet (control). ^2^ SEM—standard error mean. Note: averages do not differ between groups (*p* ≤ 0.05) or tend to differ (*p* ≤ 0.10), identified by different letters (^a,b^) on the same line.

**Table 7:** Cost per kilogram of concentrate ($/kg concentrate) and diet cost per kg of live weight gain ($/kg WG).

Variables	Experiment I ^1^		Economy, $
	Control	Treatment	
Cost per kg of Concentrate	0.38	0.33	0.04
Diet Cost per Day	1.30	1.23	0.07
Cost per kg of Weight gain ^2^	2.46 ^a^	2.09 ^b^	0.36
Cost in the experimental period/animal ^2^	67.7 ^a^	62.5 ^b^	5.28
**Variables**	**Experiment II ^1^**		
	**Control**	**Treatment**	
Cost per Kg of Concentrate	0.39	0.25	0.14
Diet Cost per Day	1.56	1.27	0.28
Cost per kg of Weight gain ^2^	2.91 ^a^	2.27 ^b^	0.63
Cost in the experimental period/animal ^2^	86.4 ^a^	68.8 ^b^	17.6

^1^ Treatments were: heifers that received a diet with cracker residue (treatment); animals that did not receive cracker residue in the diet (control). ^2^ Calculation based on diet and 20% additional expenses included. This variable were evaluated for treatment effect, using different letters to show differences between groups (*p* = 0.01). Note: average costs calculated per animal.

## Data Availability

Raw data are held by the authors and may be available upon request. The data are not publicly available due to university does not have a system to make data on published works available.

## Supplementary Materials

The following supporting information can be downloaded at: https://www.mdpi.com/article/10.3390/ani14091325/s1, Table S1: Composition of the ingredients used in the concentrate formulation; Table S2: Standardization of the analysis of volatile fatty acids in ruminal fluid; Table S3: Experiment I: Blood count of heifers fed or not with cracker residue; Table S4: Experiment II: Blood count of heifers fed or not with cracker residue.
