# Supplementary material for: Use of Cracker Residue in the Diet of Dairy Heifers: Impacts on Animal Health, Ruminal Fatty Acids Profile, Digestibility, Weight Gain, and Economic Viability
Source: Animals (Basel). 2024 Apr 29;14(9):1325. doi: 10.3390/ani14091325 (PMC11083051; doi:10.3390/ani14091325)
Supplement: Supplementary file 1 [file animals-14-01325-s001.zip › animals-2827497-supplementary.pdf]

Supplementary material Table S1: Composition of the ingredients used in the concentrate formulation.

Centesimal Composition	Corn	Cracker Residue	Soybean Bran	Wheat Bran	Soybean Hull	Soybean Oil
DM %	89.6	97.9	90.2	89.8	91.2	99.6
Ash%	1.18	1.23	6.62	4.35	4.49	-
CP %	7.6	7.7	41.7	14.53	8.15	-
EE	3.62	11.09	1.66	3.46	2.86	99.6

DM (Dry Matter), Ash (Mineral Matter), CP (Crude Protein), EE (Ether Extract).

Supplementary material Table S2: Standardization of the analysis of volatile fatty acids in ruminal fluid

	Acetic acid	Propionic acid	Butiric acid	Isovaleric acid	Valeric acid
R^2	0.9975	0.9973	0.9975	0.9971	0,9975
Equation	y = 0.0099x + 0.0057	y = 0.0176x + 0.0207	y = 0.0241x + 0.0033	y = 0.0306x + 0,0055	y = 0,0283x + 0,0105
Linear range (mmol L^{-1})*	1.06 - 168.85	1.72 - 137.27	1.32 - 105.84	0.55 - 43.81	0.55 - 43.62
LOD (mmol L^{-1})	1.06	0.86	0.66	0.27	0.27
LOQ (mmol L^{-1})	2.12	1.72	1.32	0.55	0.55
Accuracy (%)	98.30	94.89	95.82	98.29	104.26
Repeatability (RSD)	1.67	1.41	1.59	2.45	5.76

Note: * The linear range, LOD—limit of detection, and LOQ—limit of quantitation were expressed in mmol of VFA for L of ruminal fluid

Supplementary material Table S3: EXPERIMENT I: Blood count of heifers fed or not with cracker residue.

Variables	GROUPS[1]		SEM[2]	*P*-value	
	CONTROL	TREATMENT		Treat	Treat × day
Erythrocytes ($x10^6$ µL)				0.85	0.20
	7.73	7.88	0.26		
Hematocrit (%)				0.87	0.22

	39.0	38.9	0.80		
Hemoglobin (g/dL)				0.90	0.52
	10.3	10.1	0.21		
Leukocytes ($x10^3$ µL)				0.43	**0.10**
d 1	11.0	13.7	0.90		
d 15	23.4	21.6	0.88		
d 30	24.3	24.4	0.92		
d 60	21.0^{a}	17.8^{b}	0.93		
Neutrophils ($x10^3$ µL)				0.56	0.12
	5.18	4.89	0.40		
Lymphocytes ($x10^3$ µL)				0.28	0.16
	16.2	15.3	0.53		
Monocytes ($x10^3$ µL)				0.36	0.19
	1.44	1.22	0.13		
Eosinophils ($x10^3$ µL)				0.76	0.47
	0.07	0.05	0.08		

[1]Treatments were: Heifers that received a diet with cracker residue (treatment); animals that did not receive cracker residue in the diet (control). [2] SEM - Standard error mean

[a-c] Within a row, averages without a common superscript differ ($P \leq 0.05$) or tend to differ ($P \leq 0.10$).

Supplementary material Table S4: EXPERIMENT II: Blood count of heifers fed or not with cracker residue.

**Variables**	**GROUPS**[1]		**SEM**[2]	***P*-value**	
	**CONTROL**	**TREATMENT**		**Treat**	**Treat × day**
Erythrocytes ($x10^6$ µL)				0.84	0.79
	7.66	7.83	0.15		
Hematocrit (%)				0.60	0.18
	32.1	34.2	0.58		
Hemoglobin (g/dL)				0.68	0.54

	10.6	11.2	0.22		
Leukocytes (x10³ μL)				0.82	0.89
	9.05	9.14	0.52		
Neutrophils (x10³ μL)				0.30	0.21
	2.78	2.16	0.43		
Lymphocytes (x10³ μL)				0.51	0.67
	5.61	6.10	0.48		
Monocytes (x10³ μL)				0.35	0.31
	0.42	0.62	0.10		
Eosinophils (x10³ μL)				0.80	0.92
	0.12	0.11	0.04		

[1] Treatments were: Heifers that received a diet with cracker residue (treatment); animals that did not receive cracker residue in the diet (control). [2] SEM - Standard error mean

[a-c] Within a row, averages without a common superscript differ ($P \leq 0.05$) or tend to differ ($P \leq 0.10$).
